# Pancreatic Hemangioblastoma in a Patient with von Hippel-Lindau Disease: A Case Report

**DOI:** 10.70352/scrj.cr.25-0247

**Published:** 2025-09-05

**Authors:** Naoto Nakamura, Yosuke Kasai, Kazuyuki Nagai, Asahi Sato, Kentaro Kadono, Norimitsu Uza, Tsuyoshi Ohno, Sho Koyasu, Yuji Nakamoto, Noritaka Sano, Ayako Takahashi, Shinya Otsuki, Hiroaki Ito, Kei Yamane, Takayuki Anazawa, Satoshi Ogiso, Yoichiro Uchida, Takashi Ito, Takamichi Ishii, Etsuro Hatano

**Affiliations:** 1 Department of Surgery, Graduate School of Medicine, Kyoto University, Kyoto, Kyoto, Japan; 2 Department of Gastroenterological Surgery, Kyoto-Katsura Hospital, Kyoto, Kyoto, Japan; 3 VHL Center, Kyoto University Hospital, Kyoto, Kyoto, Japan; 4 Division of Gastroenterology, Department of Internal Medicine, Graduate School of Medicine, Kobe University, Kobe, Hyogo, Japan; 5 Department of Diagnostic Imaging and Nuclear Medicine, Graduate School of Medicine, Kyoto University, Kyoto, Kyoto, Japan; 6 Department of Neurosurgery, Graduate School of Medicine, Kyoto University, Kyoto, Kyoto, Japan; 7 Department of Ophthalmology and Visual Sciences, Graduate School of Medicine, Kyoto University, Kyoto, Kyoto, Japan; 8 Department of Diagnostic Pathology, Graduate School of Medicine, Kyoto University, Kyoto, Kyoto, Japan

**Keywords:** von Hippel-Lindau disease, pancreatic tumor, hemangioblastoma, duodenal hemorrhage

## Abstract

**INTRODUCTION:**

von Hippel-Lindau (VHL) disease is an autosomal dominant hereditary disorder characterized by the development of tumor-like lesions in multiple organs. While central nervous system hemangioblastomas, pancreatic neuroendocrine tumors, and pancreatic cysts are commonly associated with VHL disease, there have been few reported cases of pancreatic hemangioblastoma in patients with VHL disease.

**CASE PRESENTATION:**

A male patient in his 30s had been diagnosed with VHL disease and had been followed for cerebellar and spinal hemangioblastomas, and renal cell carcinoma, for which he had undergone several tumor resections, radiation therapy, and a ventriculoperitoneal shunt. A pancreatic head tumor deemed to be a neuroendocrine tumor on imaging findings exhibited a gradual increase in size from 12 to 33 mm for the past 2 years, but it had been monitored due to his comorbidities and declining daily living activities. Severe anemia was detected during his regular outpatient visit, and an emergency esophagogastroduodenoscopy revealed a submucosal tumor near the duodenal papilla with ulceration and active bleeding, making endoscopic hemostasis challenging. Dynamic contrast-enhanced CT showed active bleeding from the pancreatic tumor. Subsequently, emergency angiography was performed via the superior mesenteric artery, successfully embolizing vessels supplied by the inferior pancreaticoduodenal artery to achieve hemostasis. Due to concerns about rebleeding, we performed pancreaticoduodenectomy 1 month after the emergency angiography, during which we awaited the improvement of the patient’s overall condition. Microscopic findings of the tumor showed multinodular proliferation with hematoxylin-eosin staining, revealing cells with clear cytoplasm and abundant capillaries and dilated branching vessels within the nests. Immunohistochemical analysis demonstrated positivity for alpha-inhibin and S100, with partial positivity for carbonic anhydrase IX, leading to a diagnosis of pancreatic hemangioblastoma.

**CONCLUSIONS:**

This paper reports a rare case of pancreatic hemangioblastoma arising in a patient with VHL disease. It is crucial to consider the possibility of pancreatic hemangioblastoma when treating pancreatic tumors in VHL disease patients.

## Abbreviations

CA
: celiac artery
CD
: cluster of differentiation
CE-CT
: contrast-enhanced CT
CNS
: central nervous system
ECOG
: Eastern Cooperative Oncology Group
EGD
: esophagogastroduodenoscopy
IPDA
: inferior pancreaticoduodenal artery
NET
: neuroendocrine tumor
PanNET
: pancreatic neuroendocrine tumor
RCC
: renal cell carcinoma
RHA
: right hepatic artery
SMA
: superior mesenteric artery
SPN
: solid pseudopapillary neoplasm
SRS
: somatostatin receptor scintigraphy
VHL
: von Hippel-Lindau

## INTRODUCTION

VHL disease is an autosomal dominant hereditary disorder characterized by multiple tumor lesions affecting various organs. Manifestations include CNS (cerebellum, brainstem, spinal cord) hemangioblastomas, retinal hemangiomas, RCC and cysts, pheochromocytomas, epididymal cystadenomas, broad ligament cystadenomas, and intra-abdominal lymphangiomas, among others.^1–4)^ NET and serous cystadenoma are common pancreatic tumors associated with VHL disease. Reports of pancreatic hemangioblastomas are exceedingly rare, with only 2 cases documented to date,^5,6)^ and there have been no reports of pancreatic hemangioblastomas associated with VHL disease. Here, we report a rare case of pancreatic hemangioblastoma in a patient with VHL disease who underwent surgical resection.

## CASE PRESENTATION

A male patient in his 30s had been diagnosed with VHL disease (proband) 7 years prior, after the identification of cerebellar and spinal hemangioblastomas, renal cell carcinoma, and multiple pancreatic cysts. Further analysis of the VHL gene through direct sequencing of peripheral blood revealed a germline mutation, c.208G>T (p.Glu70Ter). The patient had undergone multiple tumor resections, radiation therapy, and ventriculoperitoneal shunt placement for the cerebellar and spinal hemangioblastomas, as well as partial nephrectomy for bilateral RCCs. He had been using a wheelchair, and had been having difficulty in walking alone without support. His ECOG performance status was 3 as the sequela of multiple neurosurgeries.

**Fig. 1:**
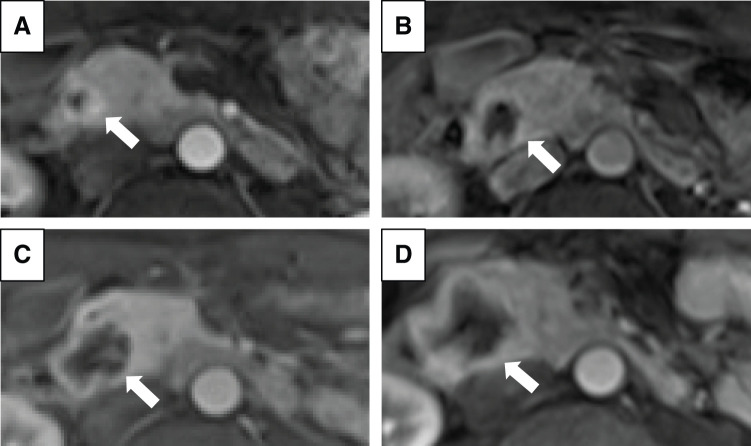
Time-dependent increase in the pancreatic tumor size on MRI. (**A**) 18 months before surgery (12 mm); (**B**) 12 months before surgery (22 mm); (**C**) 6 months before surgery (28 mm); and (**D**) immediately before surgery (33 mm). White arrows indicate the tumor.

**Fig. 2:**
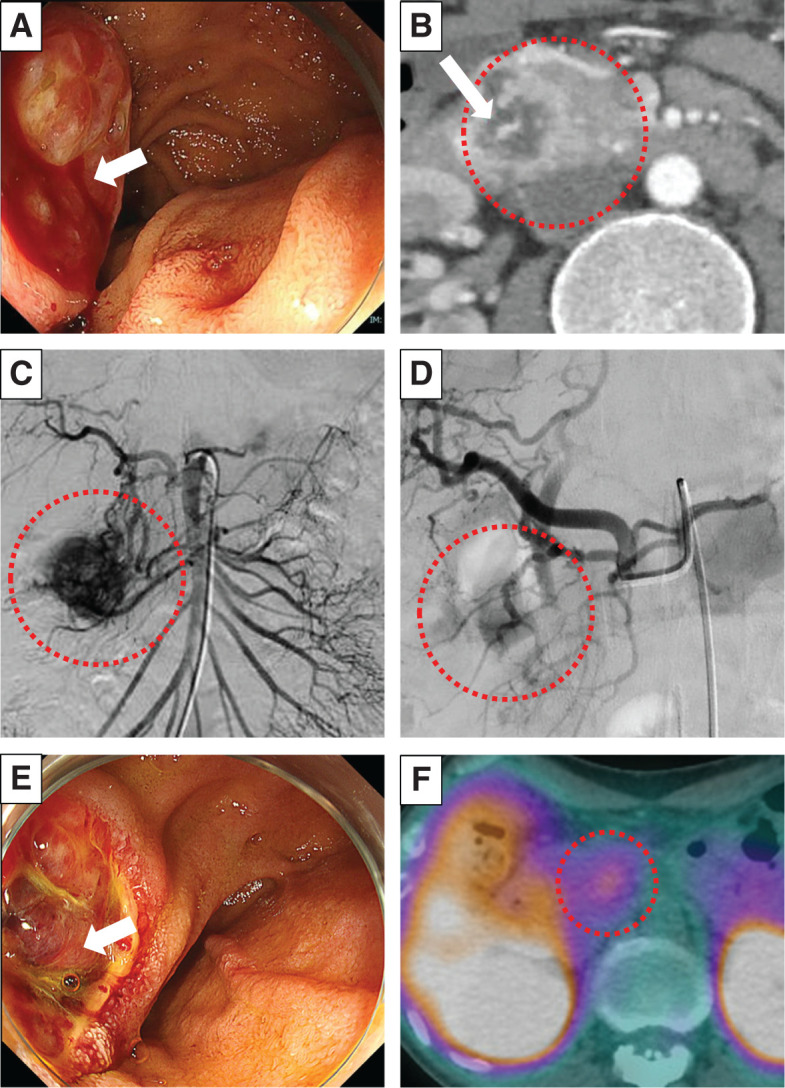
Preoperative workups. (**A**) The EGD during duodenal bleeding. A submucosal tumor with a large ulcerated apex was observed near the duodenal papilla, with active bleeding (white arrow). (**B**) Contrast-enhanced CT findings during bleeding. In the pancreatic head tumor (red dashed circle), a high-density area with contrast enhancement was observed, suggesting active bleeding (white arrow). (**C**) An emergency angiography from the SMA and CA. A dense tumor staining was observed (red dashed circle). (**D**) After embolization of the feeding arteries from SMA and CA, the tumor staining was attenuated (red dashed circle). (**E**) The EGD findings after embolization. Hemostasis was achieved, while a large blood vessel remained exposed on the ulcer surface (white arrow). (**F**) Somatostatin receptor scintigraphy showed mild uptake in the pancreatic head tumor (red dashed circle). EGD, esophagogastroduodenoscopy; CA, celiac artery; SMA, superior mesenteric artery

**Fig. 3:**
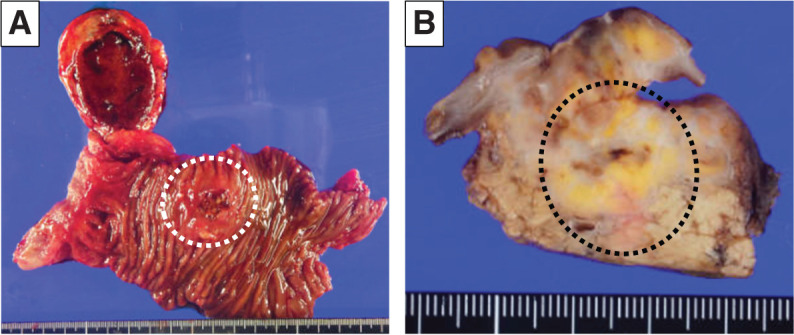
Macroscopic findings of the surgically resected specimen. (**A**) Duodenal ulceration by the pancreatic tumor (white dotted circle). (**B**) A yellowish-white, hard, 3.3 × 3.3 × 2.6 cm-sized tumor with clear boundaries in the pancreas (black dotted circle).

**Fig. 4:**
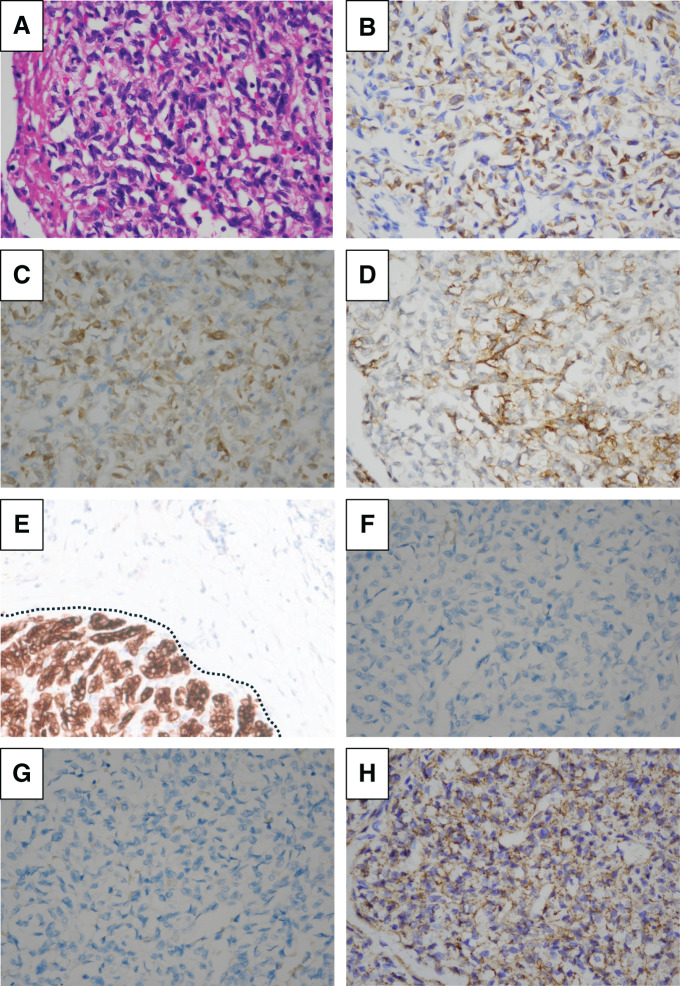
Microscopic findings of the surgically resected specimen. (**A**) The tumor cells, which had clear cytoplasm, proliferated in a multi-nodular pattern, and the tumor nodules were accompanied by abundant capillaries, and some of the blood vessels were dilated and branched in the hematoxylin and eosin-stained specimen. (**B**–**G**) The results of immunostaining were as follows: alpha-inhibin was positive (**B**), S100 was positive (**C**), carbonic anhydrase IX was partially positive (**D**), and cytokeratin was negative (The area to the right of the dashed line is hemangioblastoma, while the area to the left is normal pancreatic tissue, **E**), synaptophysin was negative (**F**), chromogranin A was negative (**G**), and β-catenin was negative for nucleus (**H**). (**A**–**H** 400×)

Two years prior, a 12-mm tumor was noted in the pancreatic head, diagnosed as a PanNET based on MRI (**Fig. 1A**). Although the tumor in the pancreatic head exhibited a gradual increase in size from 12 to 33 mm for the past 2 years (**Fig. 1B**–**1D**), it had been monitored due to his comorbidities, including active CNS hemangioblastoma and bilateral RCCs, and declining daily living activities, making surgical intervention too invasive for his condition. On a regular outpatient visit, severe anemia was detected, with a hemoglobin level of 5.5 g/dL. An emergency EGD was performed, revealing a submucosal tumor near the duodenal papilla with ulceration and active bleeding, making endoscopic hemostasis challenging (**Fig. 2A**). In dynamic CE-CT, a high-attenuation area with contrast enhancement was observed within the pancreatic head tumor from the early to late phases, suggesting hemorrhage. The low-attenuation area inside the tumor was continuous with the duodenal lumen (**Fig. 2B**). Subsequently, emergency angiography was performed via the CA and SMA, revealing tumor staining from the IPDA and the replaced RHA (**Fig. 2C**). In order to control blood flow, embolization therapy was performed on these feeding arteries, ultimately resulting in a reduction of tumor staining (**Fig. 2D**). Two days later, a repeat EGD confirmed hemostasis; however, large exposed vessels were still present, indicating a high risk of rebleeding (**Fig. 2E**). SRS revealed faint uptake in the pancreatic head tumor, compatible with PanNET with cystic degeneration (**Fig. 2F**). We performed pancreaticoduodenectomy 1 month after the emergency angiography, during which we awaited for improvement in the patient’s overall condition. The operation lasted 6 h and 42 min, with an estimated blood loss of 156 g, and no intraoperative transfusion was required. On gross findings, the resected specimen revealed a well-defined, yellowish-white, firm pancreatic mass measuring 3.3 × 3.3 × 2.6 cm with an ulcer on the duodenum (**Fig. 3A** and **3B**). Microscopically, the tumor consisted of multinodular proliferation of cells with clear cytoplasm, accompanied by abundant capillaries within the tumor nodules, with some blood vessels appearing dilated and branching (**Fig. 4A**). Immunohistochemistry of the tumor was positive for alpha-inhibin (**Fig. 4B**), S100 (**Fig. 4C**), carbonic anhydrase IX (partially, **Fig. 4D**), while it was negative for cytokeratin (**Fig. 4E**), synaptophysin (**Fig. 4F**), chromogranin A (**Fig. 4G**), or β-catenin for nucleus (**Fig. 4H**). The Ki-67 index was 15%, and no lymph node metastasis was observed. Based on these results, he was diagnosed with pancreatic hemangioblastoma. Postoperatively, no pancreatic fistula developed; however, he experienced aspiration pneumonia due to delayed gastric emptying, which improved with antibiotic treatment. He was transferred to a rehabilitation facility on POD 43. Currently, he is receiving outpatient follow-up, and no recurrence has been observed for 10 months since the surgery.

## DISCUSSION

VHL disease is an autosomal dominant hereditary disorder characterized by the development of multiple tumor lesions across various organs. Manifestations include hemangioblastomas of the CNS such as the cerebellum, medulla oblongata, and spinal cord, retinal angiomas, RCCs and renal cysts, pheochromocytomas, PanNETs and pancreatic cysts, epididymal cystadenomas, broad ligament cystadenomas, and endolymphatic sac tumors. These tumors are typically multiple, recurrent, and often present at young ages.^1–3,7)^ In 8%–17% of VHL cases, PanNETs are observed, most of which are non-functional and asymptomatic.^1–3)^ Distant metastasis is infrequent, occurring in only 11%–20% of cases at diagnosis.^8)^ Prognostic factors for VHL-associated PanNETs include: 1) maximum tumor size ≥3 cm, 2) pathogenic variant in exon 3 of the VHL gene, and 3) tumor doubling time ≤500 days.^9)^ It has been reported that distant metastasis does not occur in cases with none or only 1 of these factors, while it occurs in 33% of cases with 2 factors and in 67% of cases with all 3 factors.^10)^ The Japan NeuroEndocrine Tumor Society clinical practice guidelines for gastroenteropancreatic neuroendocrine neoplasms (2nd edition) recommend surgical intervention for tumors with a maximum diameter ≥2 cm or a tumor doubling time ≤500 days.^9)^ In the present case, the pancreatic tumor was diagnosed as PanNET based on the patient’s background and diagnostic imaging findings. Just before the hemorrhage, the tumor size had increased from 12 to 28 mm for 1 year. Although surgical intervention should have been considered based on the guidelines, conservative observation was chosen due to other comorbid tumors and the patient’s declining functional status (ECOG performance status of 3). However, the tumor’s progression led to unexpected duodenal bleeding, prompting an emergency embolization followed by an elective pancreaticoduodenectomy. As a result, the tumor was revealed to be hemangioblastoma of the pancreas.

Hemangioblastomas associated with VHL disease occur in multiple locations, including the cerebellum, medulla, pons, spinal cord, and visceral organs. It is reported that 60%–84% of VHL patients have CNS hemangioblastomas, with 50%–75% of these occurring in the cerebellum.^1,4,11)^ Hemangioblastomas are the most common tumors in VHL disease, whereas approximately 25% of hemangioblastomas are associated with VHL disease.^12–15)^ Regarding the distribution of CNS hemangioblastomas in VHL patients, a report from the National Institute of Health group in the United States showed that 51% were located in the spinal cord, 38% in the cerebellum, 10% in the brainstem, and 2% in the supratentorial region.^11)^ A Japanese hospital survey revealed that 18.9% were in the spinal cord, 71.2% in the cerebellum, 9% in the brainstem, and 0.9% in the pituitary gland.^16)^ Hemangioblastomas outside the CNS are referred to as extra-neuraxial hemangioblastomas. These are further classified into paraneuraxial and peripheral hemangioblastomas, with the latter divided into somatic (retroperitoneal or non-organic) and visceral (organ-specific), the latter of which is further subdivided into renal and non-renal.^17)^

We searched for the keywords “pancreatic hemangioblastoma” in PubMed. Among non-renal cases, excluding autopsy cases, only 2 reports of pancreatic hemangioblastoma have been documented^5,6)^ (**Table 1**). In the former case, genetic testing revealed no VHL gene mutation, but a TSC2 gene mutation (R905Q) associated with tuberous sclerosis was detected. There is no reported association between tuberous sclerosis and pancreatic hemangioblastomas, thus the case was considered potentially confidential.^5)^ In the latter case reported in 1966, preoperative imaging showed only gastric compression on gastrointestinal fluoroscopy. It remained uncertain whether it was actually a hemangioblastoma because of the lack of verifiable evidence.^6)^

**Table 1:** Analysis of reported cases of pancreatic hemangioblastoma

No	Author/Year	Sex/Age	Subsequent genetic testing for VHL	Complaint or Symptom	Tumor location	Tumor size	Surgical method	Immunostaining
1	Perez et al.^5)^/2024	M/45	Negative	None (hyperbilirubinemia)	Pancreatic head	Not specified	PD	(+) CD34, S100 and inhibin
2	Shirakabe et al.^6)^/1966	F/26	Not specified	Epigastric fullness	Pancreatic tail	Not specified	DP	Not performed
3	Our case	M/39	Positive	Anemia detected in blood test	Pancreatic head	33 mm	PD	(+) inhibin, S100, carbonic anhydrase IX
								(−) Cytokeratin, Synaptophysin, Chromogranin A, β-catenin

CD34, cluster of differentiation 34; DP, distal pancreatectomy; F, female; M, male; PD, pancreaticoduodenectomy; VHL, von Hippel-Lindau

Retrospectively, it would have been extremely difficult to consider hemangioblastoma as a differential diagnosis for a pancreatic mass—regardless of whether the patient had VHL disease or not. Given the extreme rarity of pancreatic hemangioblastoma and the near-total absence of relevant imaging reports in the literature, differential diagnosis in this setting is inherently limited. As for the possibility of extrapolating from the imaging features of CNS hemangioblastoma, that would also have been difficult. Most VHL-associated hemangioblastoma present as solid, non-necrotic nodules, either solitary or accompanied by cysts. In this respect, the pancreatic lesion in our case showed few similarities, and it would have been highly challenging to identify it as a hemangioblastoma preoperatively. Regarding the interpretation of the SRS, in a necrotic nodule—especially one assessed after embolization—it is virtually impossible to rule out a NET, regardless of the degree of tracer uptake. Low uptake is not uncommon in G3 NETs, which further limits the interpretability of SRS under such conditions. If one suggestive finding were to be highlighted retrospectively, it would be the marked hypervascularity seen on angiography. This degree of vascularity may have exceeded that typically seen in PanNETs and could have suggested the hypervascular nature of hemangioblastoma. Nonetheless, given the emergent context in which embolization was performed, it is understandable that there was no realistic opportunity at that time to reconsider such a rare and specific differential diagnosis.

Histopathological features of hemangioblastomas include the presence of spindle cells with pleomorphism and vacuolated cytoplasm, occurring in vascular-rich areas, as well as areas poor in vessels but rich in inflammatory cells and elastic fibers.^18)^ The pathology of our case matched these characteristics. Immunohistochemical staining of hemangioblastoma was typically positive for vimentin, carbonic anhydrase, S100 protein, neuron-specific enolase, CD57, and inhibin, and negative for glial fibrillary acidic protein, cytokeratin, epithelial membrane antigen, CD34, HMB-45, desmin, and actin.^18)^ The combination of negative cytokeratin and positive carbonic anhydrase IX is highly sensitive (90%) and specific (100%) for the diagnosis of hemangioblastoma.^19)^ These features were present in this case. Differential diagnoses included clear cell RCC, PanNET, and SPN. However, clear cell RCCs typically show positivity for cytokeratin and PAX8; PanNETs are generally positive for synaptophysin, chromogranin A, and cytokeratin; and SPNs characteristically exhibit nuclear positivity for β-catenin. These features were not consistent with those observed in our case.

Although endoscopic ultrasound-guided fine needle aspiration/biopsy might have been useful for preoperative diagnosis of pancreatic hemangioblastoma, it was challenging for this patient due to his poor condition and the duodenal hemorrhage. Alternatively, SRS was performed, which showed undetermined faint uptake in the tumor. In retrospect, the relatively rapid growth of the tumor and the duodenal hemorrhage were atypical for the usual clinical course of PanNETs. These findings suggest that there might have been an opportunity to consider alternative differential diagnoses. Nevertheless, it would have been virtually impossible to suspect hemangioblastoma, given the exceedingly rare incidence even among patients with VHL disease, as mentioned above. Regardless of the diagnosis, pancreaticoduodenectomy would have been inevitable due to the concerns over rebleeding.

## CONCLUSIONS

The pancreatic hemangioblastoma presented in this report was challenging to diagnose through imaging alone. In this case, PanNET was initially suspected based on imaging, and careful follow-up was conducted. Although hemangioblastomas are generally considered as benign tumors, as in this case, rapid growth and tumor hemorrhage can occur, leading to life-threatening outcomes. In this patient, hemostasis was achieved through prompt vascular embolization, enabling an elective surgery. When differentiating pancreatic tumors in patients with VHL disease, the possibility of hemangioblastoma should also be considered. Accumulation of more cases is needed to facilitate smoother diagnosis and treatment in the future.
